# Adverse reaction to food screening test: new multiparametric ELISA kit for patients with gastrointestinal symptomatology linked to ingesting food

**DOI:** 10.1186/1939-4551-8-S1-A243

**Published:** 2015-04-08

**Authors:** Diego Faggian, Flaviano Favaro, Isabella Solinas, Mario Plebani

**Affiliations:** 1 University Hospital of Padua, Italy; 2 AMS S.p.a. Analyzer Medical System, Italy

## Background

An adverse reaction to food is a general term describing clinically abnormal responses to an ingested food that might occur secondary to non-allergic food hypersensitivity (food intolerance) or allergic food hypersensitivity (food allergy). It is well known that almost 1 person in 4 suffers from an intestinal complaint, due to this fact different kits have been put on the market, generally with no scientific validity and inadequate diagnostic effect. This test is an enzyme immunoassay screening kit for single patients who present symptoms in the gastrointestinal tract. This is an initial screening test that should facilitate the diagnosis of adverse reactions to food (food allergy, food intolerance and celiac disease), or at least the exclusion of these possibilities.

## Methods

The kit, a microplate streptavidin coated, provides the semi-quantitative determination through six different mixes of biotinylated allergens that bind to the specific IgEs (food allergy) and specific IgG_4_s (food intolerance) present in the patients' serum for the following food groups: 1) milk-eggs-meat 2) fish-shellfish 3) nuts 4) cereals 5) vegetables 6) fresh fruit, in total 41 allergens. Plus a seventh dosage mix for deaminated gliadin IgG (celiac disease).

## Results

The correspondence among mix results and the single allergens using 50 serum samples known as IgEs and IgG4s has been confirmed. This correspondence turned out to be >92% for the IgEs and >95% for the IgG_4_s. As for the comparison between the deaminated gliadin mix with 80 serum samples known as anti-transglutaminase IgA, the result was >94%.

## Conclusion

The adverse reaction to food screening test appears to be a kit providing an innovative approach for single patients, with interesting clinical applications and considerable savings from an economic point of view. Moreover it reduces invasiveness for the patient due to an evident lower requirement of serum. Therefore starting from the idea that some intestinal pathologies could be made better by excluding some foods from the patient's daily diet, this test could be an initial and also significant approach in the search for foods which may provoke gastrointestinal symptoms in patients.

